# Simultaneous Localization and Mapping with Iterative Sparse Extended Information Filter for Autonomous Vehicles

**DOI:** 10.3390/s150819852

**Published:** 2015-08-13

**Authors:** Bo He, Yang Liu, Diya Dong, Yue Shen, Tianhong Yan, Rui Nian

**Affiliations:** 1 School of Information Science and Engineering, Ocean University of China, 238 Songling Road, Qingdao 266100, China; E-Mails: bhe@ouc.edu.cn (B.H.); oucliuyang@126.com (Y.L.); dongdiya007@163.com (D.D.); nianrui_80@163.com (R.N.); 2 School of Mechanical and Electrical Engineering, China Jiliang University, 258 Xueyuan Street, Xiasha High-Edu Park, Hangzhou 310018, China; E-Mail: yanth@163.com

**Keywords:** autonomous vehicles, autonomous navigation, SLAM, SEIF, consistency, scalability, iteration

## Abstract

In this paper, a novel iterative sparse extended information filter (ISEIF) was proposed to solve the simultaneous localization and mapping problem (SLAM), which is very crucial for autonomous vehicles. The proposed algorithm solves the measurement update equations with iterative methods adaptively to reduce linearization errors. With the scalability advantage being kept, the consistency and accuracy of SEIF is improved. Simulations and practical experiments were carried out with both a land car benchmark and an autonomous underwater vehicle. Comparisons between iterative SEIF (ISEIF), standard EKF and SEIF are presented. All of the results convincingly show that ISEIF yields more consistent and accurate estimates compared to SEIF and preserves the scalability advantage over EKF, as well.

## 1. Introduction

Autonomous navigation in environments without prior knowledge is a crucial problem for various autonomous vehicles [1,2,3]. The inertial navigation system, also known as INS, had been widely used to provide position estimates. However, the estimate error of the INS grows with time unboundedly, resulting in an inaccurate estimate ultimately. GPS can be utilized as an auxiliary means to modify the estimate in some applications. Unfortunately, the GPS signal is not available in some common environments (e.g., indoor and underwater), which requires an alternative method to implement accurate navigation.

Many SLAM algorithms have been proposed based on the probabilistic formulation of the problem [4] in the past few years. Among the SLAM algorithms, EKF-based SLAM shows its competency to solve the problem. Due to the simplicity of the algorithm, EKF SLAM has been widely used in many applications [5,6]. However, the shortcomings of EKF SLAM are also exposed at the same time. The computational cost of the algorithm is quadratic to the number of features in the environments, making the online application in large-scale environments impossible [7]. In addition, in order to deal with the nonlinearity, EKF SLAM expands the nonlinear function in Taylor series and uses the first order to make an approximation. The truncation error of the approximation leads to inconsistent and inaccurate state estimation.

A number of improved methods are then proposed to overcome the weakness of EKF SLAM, and some work focuses on the scalability limitation. Among them, the information-based filter proves to be effective to deal with large-scale environments. Instead of a mean vector and a dense covariance matrix, the extended information filter (EIF) adopts the information matrix and information vector to describe the SLAM distribution [8]. Since the complexity of EIF is even greater than the EKF, it seems that EIF is not capable of solving the scalability problem as well. However, a novel insight reveals that the information matrix is sparse in the context of feature-based SLAM. Taking advantage of the sparseness, the sparse extended information filter (SEIF) was then presented [9,10]. By pruning weak links in the information matrix, SEIF keeps the information matrix sparse all of the time, thus allowing all of the updates to be executed in constant time, regardless of the size of the environment.

Another kind of SLAM algorithm is based on particle filters [11,12]. The particle filter is a kind of Monte Carlo method applying samples to present the probability density distribution, which can be employed to any state models, and when the sample size tends to be infinite, it can approximate any form of probability density distribution. FastSLAM is one of this kind of algorithm [13,14]. It can deal with multi-hypothesis association problems because of the independence of the particles. According to Tim Bailey *et al.* [15], the FastSLAM algorithm cannot guarantee the consistency of the robot pose estimation. In addition, increasing the number of particles can improve the consistency to a certain extent, but it will also increase the computational cost.

SEIF SLAM has been demonstrated to be efficient and scalable in real-world applications [16,17]. However, as a dual form of EKF, SEIF suffers from linearization errors, like EKF, as well, which may result in inaccuracy and inconsistency [15]. Moreover, due to the elimination of weak links in the sparsification step and the state recovery step, SEIF can even more easily be divergent than the EKF algorithm [18]. To refine the estimate results of SEIF, some approaches have been designed, and one of them is the exactly sparse extended information filter (ESEIF) proposed by Matthew Walter *et al.* [19]. ESEIF keeps exact sparseness by marginalizing out the robot pose occasionally from the distribution and then relocalizing the robot, which can maintain conservative pose and map estimates. ESEIF also has been successfully implemented in some real-world applications [20,21]. However, compared to SEIF, ESEIF also suffers from the same problem as EKF for nonlinear applications. Ryan M. Eustice *et al.* [22] proposed an exactly sparse delayed-state filter (ESDSF). It is based on a delayed-state framework in which the information matrix is exactly sparse. This framework can be used in view-based representation of the environments, which rely on scan matching sensor data; while in feature-based environments, it is not applicable anymore.

Iteration is an effective method in nonlinear estimation problems and has been demonstrated to be effective in SLAM algorithms [23,24,25]. The iterated extended Kalman filter (IEKF) is one of the most popular iterated filters [26]. IEKF achieves better consistency and accuracy compared to the original EKF. However, the learning speed of IEKF is even slower than EKF, which limits its applications. In this paper, we investigate the role of iteration in the SEIF SLAM algorithm and then propose an iterative sparse extended information filter (ISEIF). We will first introduce the reasons that lead to the inconsistency in the SEIF SLAM algorithm. Then, we will derive the iterative form of SEIF and illustrate how it works. Theoretical analysis will be given to prove the consistency and accuracy, and the scalability will be considered, as well. To the best of our knowledge, this is the first time information filters have been refined through iterative methods.

To demonstrate the advantages of the ISEIF algorithm, Monte Carlo simulation results will be first presented. After that, the application of the ISEIF SLAM algorithm to autonomous vehicles, including an autonomous ground vehicle (AGV) and an autonomous underwater vehicle (AUV), will be studied. The Victoria Park dataset was collected by an autonomous ground vehicle, which was developed at the Australian Center for Field Robotics (ACFR) at the University of Sydney [27]. It carries a number of essential sensors, such as a velocity encoder, a steering angle sensor, an inertial measurement unit, GPS, a laser and a vision sensor. We use this dataset to demonstrate the ability of autonomous outdoor navigation for ISEIF SLAM in large-scale unstructured environments. The C-Ranger AUV platform was developed at the Underwater Vehicle Laboratory at Ocean University of China [28,29], which is equipped with a variety of sensors, such as GPS, attitude and heading reference system (AHRS), digital compass, gyro, Doppler velocity log (DVL) and multibeam imaging sonar for active sensing in underwater environments. An output feedback controller and motion planning algorithm is adopted to achieve the task of path following [30,31]. We will also verify the feasibility of ISEIF SLAM for the C-Ranger AUV by sea trials in Tuandao Bay. Experimental results will show that compared to SEIF, ISEIF performs a more conservative accurate estimate and also preserves the scalability advantage over EKF SLAM.

The remainder of the paper is organized as follows: in Section 2, we formulate the basic equations of iterative SEIF with detailed theoretical analysis. Both simulated and experimental results will be presented to verify the superiority of the proposed algorithm in Section 3. Finally, we have a discussion of the ISEIF SLAM algorithm and draw the conclusion.

## 2. The Iterative SEIF

As an alternative form of EKF, EIF suffers from the same statistical and analytical linearization error propagation, which may cause inconsistency in SLAM. Additionally, due to some unique steps (*i.e.*, sparsification and state recovery) in SEIF, the consistency and accuracy of SEIF is even inferior to EKF algorithm. In this section, we will first review the basic steps of SEIF. The causes of inconsistency and inaccuracy existing in SEIF will then be analyzed. After the analysis, an adaptive ISEIF is proposed for the purpose of improving the consistency and accuracy of SEIF.

### 2.1. Review of SEIF

The information form of the SLAM problem is as follows.

Let $\xi_{t}$ denote the state vector at time *t*, which includes the robot pose $x_{t}$ and the set of features location $\left\{y_{1},\ldots ,y_{n}\right\}$:

(1) $$\xi_{t}={\left(x_{t},y_{1},\ldots ,y_{n}\right)}^{T}$$

In SLAM problem, the robot aims to find a probabilistic estimate of $\xi_{t}$. Following the Bayesian rule, the estimation problem transforms to calculate a posterior distribution $p(\xi_{t}|z^{t},u^{t})$, where $z^{t}$ and $u^{t}$ are the observations and control inputs at time *t*, respectively. Reviewing the EKF solution to the SLAM problem, the posterior $p(\xi_{t}|z^{t},u^{t})$ is represented by the mean $\mu_{t}$ and the covariance matrix $\Sigma_{t}$:

(2) $$p\left(\xi_{t}|z^{t},u^{t}\right)\propto exp\left\{-\frac{1}{2}{\left(\xi_{t}-\mu_{t}\right)}^{T}\Sigma_{t}^{-1}\left(\xi_{t}-\mu_{t}\right)\right\}$$

Instead of $\mu_{t}$ and $\sum_{t}$, information filters represent the posterior through $H_{t}$ and $b_{t}$, which are the information matrix and information vector, respectively. They are defined as follows:

(3) $$H_{t}=\Sigma_{t}^{-1}$$

(4) $$b_{t}=\mu_{t}^{T}H_{t}$$

Then, the posterior can be represented in the commonly-known information form of the Kalman filter:

(5) $$p\left(\xi_{t}|z^{t},u^{t}\right)\propto exp\left\{-\frac{1}{2}\xi_{t}^{T}H_{t}\xi_{t}+b_{t}\xi_{t}\right\}$$

The measurement model is:

(6) $$z_{t}=h\left(\xi_{t}\right)+\varepsilon_{t}$$

The measurement $z_{t}$ is usually governed by a deterministic nonlinear function *h* of the state vector $\xi_{t}$, where the term $\varepsilon_{t}$ represents independent Gaussian noise.

In addition to the measurement update step, SEIF also needs to perform a motion update, a sparsification step and a state recovery step to solve the SLAM problem.

### 2.2. Consistency Analysis of SEIF

The SEIF SLAM shows poor consistency compared to the traditional EKF SLAM algorithm, which results from approximations. There are three approximation steps existing in SEIF:

First, SEIF approximated the nonlinear function in SLAM by first order Taylor series. It deals with the nonlinear measurement model by expanding it in a Taylor series around $\mu_{t}$ and uses the following equation to make an approximation:

(7) $$h\left(\xi_{t}\right)\approx h\left(\mu_{t}\right)+C_{t}^{T}\left[\xi_{t}-\mu_{t}\right]$$

where $C_{t}$ is the Jacobian of *h* with respect to $\mu_{t}$.

Based on the linearization, we can then update the measurement equations [9].

**Figure 1:**
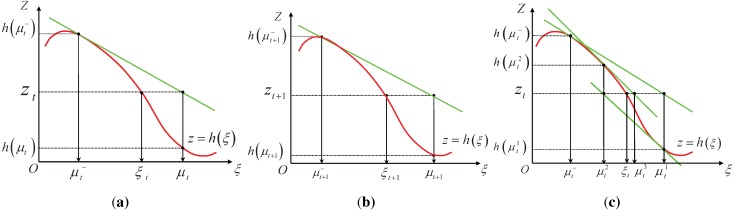
(**a**) Estimate error at time *t*; (**b**) estimate error at time t+1; and (**c**) the iterative process.

After the measurement update equations are executed, we obtain the posterior estimate $\mu_{t}$. However, as illustrated in Figure 1a, the pose estimate $\mu_{t}$ is still far away from the true state $\xi_{t}$, which results in estimate error. As the robot moves on, the next pose prediction $\mu_{t+1}^{-}$, which is calculated based on the previous estimate $\mu_{t}$; the motion model *h* is even further to the true pose $\xi_{t+1}$; and the estimate result $\mu_{t+1}$ after measurement update equations is still further to the true state $\xi_{t+1}$, as illustrated in Figure 1b. The result shows that error will accumulate over time.

Second, SEIF only maintains the information vector and the information matrix, while we also need the estimated state vector during some steps. SEIF gets the state vector by the state recovery step, which is formulated as an optimization problem; hence, the recovery of the state is also an approximation, and this leads to estimation error, as well.

Last, the information matrix is not naturally sparse in the calculating process; SEIF prunes the weak links in the matrix to maintain the sparseness of the information matrix. Therefore, this is actually an approximation, as well, which also leads to estimation error.

Due to the three approximation steps, SEIF tends to be more inconsistent compared to traditional EKF SLAM, although it keeps a very fast speed. Among the three reasons, the first one is the largest contribution to the inconsistency. Therefore, we will focus on reducing the linearization error to improve the consistency of SEIF.

### 2.3. The Adaptive ISEIF

The modified measurement update equations can be obtained by an iterative method, which is termed the iterative sparse extended information filter. Instead of updating the state as a linear combination of the prediction and the innovation, we solve it as a maximum *a posteriori* (MAP) problem. The estimate of the MAP problem can be obtained by iteratively relinearizing the measurement equation.

The conditional probability density function (pdf) of $\xi_{t}$ given $Z^{t+1}$ can be written as:

(8) $$\begin{array}{cc} p[\left.\xi (t+1)\right|Z^{t+1}] & =p[\left.\xi (t+1)\right|z\left(t+1\right),Z^{t}]\\ & =\frac{1}{c}p\left[\left.z(t+1)\right|\xi \left(t+1\right)\right]p\left[\left.\xi (t+1)\right|Z^{t}\right]\\ & =\frac{1}{c}ℵ\left[z\left(t+1\right);h\left[t+1,\xi \left(t+1\right)\right],R\left(t+1\right)\right]\\ & \;\;\;\;\;\cdot ℵ[\xi \left(t+1\right);\mu \left(\left.t+1\right|t\right),P\left(\left.t+1\right|t\right)] \end{array}$$

Maximizing the above equations is equivalent to minimizing the following equations:

(9) $$\begin{array}{cc} J[\xi (t+1)]= & \frac{1}{2}{\left\{z\left(t+1\right)-h\left[t+1,\xi \left(t+1\right)\right]\right\}}^{\prime }R{\left(t+1\right)}^{-1}\\ & \cdot \{z(t+1)-h[t+1,\xi (t+1)]\}\\ & +\frac{1}{2}{\left[\xi \left(t+1\right)-\mu \left(\left.t+1\right|t\right)\right]}^{\prime }P{\left(\left.t+1\right|t\right)}^{-1}\\ & \cdot [\xi \left(t+1\right)-\mu \left(\left.t+1\right|t\right)] \end{array}$$

*J* can be expanded in a second order Taylor series, and we could use the Newton–Raphson algorithm to get the MAP estimate of $\xi_{t+1}$:

(10) $$J=J^{i}+J{{}_{\xi }^{i}}^{\prime }\left(\xi_{t+1}-\mu_{t+1}^{i}\right)+\frac{1}{2}{\left(\xi_{t+1}-\mu_{t+1}^{i}\right)}^{\prime }J_{\xi \xi }^{i}\left(\xi_{t+1}-\mu_{t+1}^{i}\right)$$

where:

(11) $$J^{i}={\left.J\right|}_{\xi =\mu_{t+1}^{i}}$$

(12) $$J_{\xi }^{i}=\nabla_{\xi }{\left.J\right|}_{\xi =\mu_{t+1}^{i}}$$

(13) $$J_{\xi \xi }^{i}=\nabla_{\xi }\nabla_{\xi }^{\prime }{\left.J\right|}_{\xi =\mu_{t+1}^{i}}$$

are the gradient and Hessian matrix of *J* with respect to $\xi_{t+1}$.

Setting the Equation (10) with respect to $\xi_{t+1}$ to zero yields the next value of $\mu_{t+1}$ in the iteration:

(14) $$\mu_{t+1}^{i+1}=\mu_{t+1}^{i}-{\left(J_{\xi \xi }^{i}\right)}^{-1}J_{\xi }^{i}$$

Then, the following three equations are iteratively calculated:

(15) $$H_{t+1}^{i}\overset{\Delta }{\;=}h_{\mu }\left[t+1,\mu^{i}\left(\left.t+1\right|t+1\right)\right]$$

(16) $$\begin{array}{cc} \mu^{i+1}\left(\left.t+1\right|t+1\right)= & \mu^{i}\left(\left.t+1\right|t+1\right)+P^{i}\left(\left.t+1\right|t+1\right){H_{t+1}^{i}}^{\prime }\\ & \cdot R{\left(t+1\right)}^{-1}\left\{z\left(t+1\right)-h\left[t+1,\mu^{i}\left(\left.t+1\right|t+1\right)\right]\right\}\\ & -P^{i}\left(\left.t+1\right|t+1\right)P{\left(\left.t+1\right|t\right)}^{-1}\\ & \cdot [\mu^{i}\left(\left.t+1\right|t+1\right)-\mu \left(\left.t+1\right|t\right)] \end{array}$$

(17) $$\begin{array}{cc} P^{i}\left(\left.t+1\right|t+1\right)= & P\left(\left.t+1\right|t\right)-P\left(\left.t+1\right|t\right){H_{t+1}^{i}}^{\prime }\\ & \cdot {\left[H_{t+1}^{i}P\left(\left.t+1\right|t\right){H_{t+1}^{i}}^{\prime }+R\left(t+1\right)\right]}^{-1}\\ & \cdot H_{t+1}^{i}P\left(\left.t+1\right|t\right) \end{array}$$

Since we get the iterative form of state estimate $\mu_{t}$, we can then calculate the information matrix and information vector iteratively:

(18) $$H_{t}=H_{t}^{i}+C_{t}^{i+1}R_{t}^{-1}{\left(C_{t}^{i+1}\right)}^{T}$$

(19) $$b_{t}^{i+1}=b_{t}^{i}+{\left(z_{t}-{\hat{z}}_{t}+{\left(C_{t}^{i+1}\right)}^{T}\mu_{t}^{i+1}\right)}^{T}R_{t}^{-1}{\left(C_{t}^{i+1}\right)}^{T}$$

where $C_{t+1}^{i}$ is the Jacobian matrix of *h* at $\mu_{t}^{i+1}$.

Figure 1c illustrates the relinearization of the nonlinear measurement function $h(\xi_{t})$ for three iterations. As shown in the figure, each new estimate can be obtained by the true measurement $z_{t}$ and the approximated function around the previous estimate. We can specify the number of iterations we want to perform in advance according to the designer’s experience. It should be noticed that the iterative form of SEIF only relinearizes the measurement model.

In fact, the estimate uncertainty is small when the robot starts to move on, and it grows with time. Therefore, it is unnecessary to perform the iterations at each step. In order to decrease the computational cost of ISEIF, the iteration will be performed periodically. The periods are usually set as 100 or 500. However, the number of iterations at each time is still difficult to choose, *i.e.*, if the number is too big, the algorithm will become slow, and the ISEIF will lose its speed advantage; on the other hand, if the number is too small, the estimate error may still be large, and the result still tends to be inconsistent. We apply an adaptive stop criteria to the iteration process to balance the complexity and consistency. The iteration stop criteria is defined as follows:

$$delta=g(\mu_{t}^{i}-\mu_{t}^{i-1})$$

where *g* is a function of the difference between two iterations (usually the distance between $\mu_{t}^{i}$ and $\mu_{t}^{i-1}$), and we will compare delta with a previous set threshold. If delta is bigger than the threshold, we will continue the iteration process, for we can still improve the estimate accuracy by iteration; and if delta is smaller than the threshold, the iteration process will be terminated. This adaptive stop criteria provides a good balance between complexity and consistency. The value of the threshold is usually determined by the designer’s experience. If delta is set properly, the ISEIF algorithm will only perform two or three iterations most of the time.

Let $\mu_{t}^{i}$ denote the posterior estimate of state $\xi_{t}$ after *i* iterations have been performed. Therefore, $\mu_{t}^{0}$ refers to the predicted estimate that derives from the motion update step in standard SEIF. We use $H_{t}^{i}$ and $b_{t}^{i}$ to denote the intermediate information matrix and information vector at the *i*-th iteration respectively, and $C_{t}^{i}$ to refer to the Jacobian of $h(\xi_{t})$ with respect to $\mu_{t}^{i}$.

With these notations, the adaptive ISEIF can then be summarized as follows:

1. The nonlinear motion and measurement model functions are given as follows:
  (20) $$\begin{array}{c} x_{t}=f\left(x_{t-1},u_{t}\right)+v_{t} \end{array}$$
  (21) $$\begin{array}{c} z_{t}=h\left(\xi_{t}\right)+\varepsilon_{t} \end{array}$$
  $$v_{t}∼\left(0,Q_{t}\right)$$
  $$\varepsilon_{t}∼\left(0,Z_{t}\right)$$
2. Initialization as follows:
  (22) $$\begin{array}{c} \mu_{0}=E\left(\xi_{0}\right) \end{array}$$
  (23) $$\begin{array}{c} P_{0}=E\left[\left(\xi_{0}-\mu_{0}\right){\left(\xi_{0}-\mu_{0}\right)}^{T}\right] \end{array}$$
  (24) $$\begin{array}{c} H_{0}=P_{0}^{-} \end{array}$$
  (25) $$\begin{array}{c} b_{0}=H_{0}*\mu_{0} \end{array}$$
3. For t=1,2,⋯, do the following:
  (a) Perform motion update equations of SEIF. The iterative SEIF is the same as the SEIF up to this point.
  (b) If *t* is not in the iterative periods, skip to d; else start to perform iterative steps as follows: Initialize the iterative SEIF to the standard SEIF and perform the iterative measurement update step.
    (26) $$\begin{array}{c} H_{t}^{0}=H_{t} \end{array}$$
    (27) $$\begin{array}{c} b_{t}^{0}=b_{t} \end{array}$$
    Calculate the measurement update Equations (14) to (18).
  (c) Calculate the value of delta:
    (28) $$delta=g(\mu_{t}^{i}-\mu_{t}^{i-1})$$
    According to the adaptive stop criteria, if delta is smaller than the threshold, stop the iteration process and turn to d; otherwise, return to the b step to continue the iteration.
  (d) The final information matrix and information vector are given as follows:
    (29) $$\begin{array}{c} H_{t}=H_{t}^{n+1} \end{array}$$
    (30) $$\begin{array}{c} b_{t}=b_{t}^{n+1} \end{array}$$
    where *n* is the number of iterations.
4. Perform the sparsification step of SEIF.

## 3. Experiments

Simulations, the Victoria Park dataset and our sea trial experiments are carried out to verify the algorithm. We use the RMSE error and normalized estimation error square (NEES) value to evaluate the accuracy and consistency and the elapsed time to evaluate the complexity. RMSE and NEES are two commonly-used metrics in the SLAM community, and they are calculated based on the ground truth data. It should be noted that relative error and other metrics are indeed very popular in the recent robotics community. Surely, they are very useful to evaluate a SLAM algorithm by itself.

However, this paper focuses more on engineering applications, not only the SLAM algorithm itself. In this paper, we try to improve the SEIF algorithm and, more importantly, implement the ISEIF algorithm for our AUV system to verify the navigation algorithm. It is hard to say that a SLAM algorithm with low relative error will have good navigation accuracy. In this situation, we care more about the navigation accuracy calculated by the navigation system in real time compared to ground truth data (e.g., GPS data). Therefore, we think it might be proper to compare the calculated trajectory with the ground truth data in this application.

### 3.1. Simulations

In this section, the simulated experiments are implemented to compare the performance of the methods described above. Fifty Monte Carlo simulations with both SEIF and ISEIF are conducted respectively under the same environments and the same basic parameters. The entire experiments are on the foundation of the geometric feature map. We use $\left(x_{t},y_{t},\varphi_{t}\right)$ to denote the vehicle pose, in which $\left(x_{t},y_{t}\right)$ are the coordinates and $\varphi_{t}$ stands for the orientation on the basis of the map environment. Given the true position of the vehicle and the coordinates of all landmarks, the expressions of the motion model are formulated as follows:

(31) $$\left[\begin{array}{c} x_{t}\\ y_{t}\\ \varphi_{t} \end{array}\right]=\left[\begin{array}{c} x_{t-1}+v_{t}\times dt\times \cos\left(\varphi_{t-1}+G\right)\\ y_{t-1}+v_{t}\times dt\times \sin\left(\varphi_{t-1}+G\right)\\ \varphi_{t-1}+v_{t}\times dt\times \sin\left(G\right)/B \end{array}\right]+W_{t}$$

where $u=[v_{t},G]$ is the control input, in which $v_{t}$ is the velocity and *G* is the steer input. In addition, *B* corresponds to the wheelbase of the vehicle, and $W_{t}$ is Gaussian process noise. The observation model is:

(32) $$\left[\begin{array}{c} r\\ b \end{array}\right]=\left[\begin{array}{c} \sqrt{{\left(m_{x}-x_{t}\right)}^{2}+{\left(m_{y}-y_{t}\right)}^{2}}\\ \tan^{-1}\left(\frac{m_{y}-y_{t}}{m_{x}-x_{t}}\right)-\varphi_{t} \end{array}\right]+N_{t}$$

where $\left(m_{x},m_{y}\right)$ are the positions of landmarks and $N_{t}$ is the observation noise. The observation model we use is the vector $\left(r,b\right)$, where *r* and *b* are the range and bearing between the landmark and the vehicle, respectively. In terms of the map, a circle trajectory composed of 20 points is designed as the waypoints, and 200 landmarks lie astride the waypoints, which form two circles, as well.

All experiments are carried out on the platform of MATLAB. The vehicle is initialized and starts at $\left(0,0\right)$. The other essential parameters in the algorithm settings are listed at Table 1.

**Table 1:** Basic parameters set in the simulation.

Wheelbase of vehicle	4 m	Control noise	(0.3 m/s, $2^{\circ }$)
Speed	3 m/s	Observation noise	(0.2 m/s, $2^{\circ }$)
Maximum steering angle	$30^{\circ }$	Control frequency	40 Hz
Maximum range	30 m	Observation frequency	5 Hz

**Figure 2:**
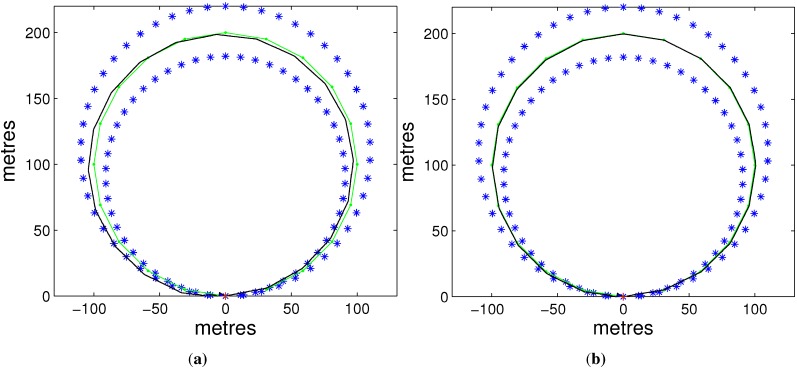
The results of trajectory and landmarks estimated by SEIF (**a**) and iterative SEIF (ISEIF) (**b**).

The ultimate data we analyze come from the average result of 50 Monte Carlo simulations. We will demonstrate the superiority of ISEIF from the following aspects: RMS, NEES and time. Figure 2 presents the estimate results of vehicle’s pose and landmarks of SEIF and ISEIF, where we can see that ISEIF has a more accurate estimate of the vehicle’s position.

**Figure 3:**
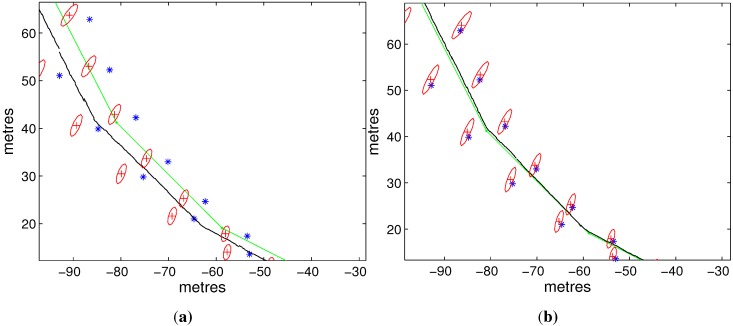
The enlarged portions of the local map, in which (**a**) is for SEIF and (**b**) is for ISEIF.

Figure 3 is a magnified local map that indicates the detailed relations between the estimates and the true position. The ellipses reflect the estimated covariances of the corresponding methods, which denote the estimate uncertainty. As the results illustrate, the estimate result of ISEIF is closer to the true landmarks in the map; and what is more important is that the estimate uncertainty in SEIF is smaller than the real error, *i.e.*, “overconfidence”, which is the main cause of inconsistency. In contrast, ISEIF performs a consistent estimate most of the time.

We use two different methods to compare the performance in different perspectives, namely we use root mean square (RMS) and normalized estimation error square (NEES) for the comparison of accuracy and consistency, respectively. They are defined as follows.

The RMS is defined as:

(33) $$e=\sqrt{\frac{\sum_{i=1}^{n_{x}}{\left(x_{i}-{\hat{x}}_{i}\right)}^{2}}{n_{x}}}$$

The NEES is defined as:

(34) $$\varepsilon_{i}={\left(x_{i}-{\hat{x}}_{i}\right)}^{T}P_{i}^{-1}\left(x_{i}-{\hat{x}}_{i}\right)$$

where $x_{i}$ is the ground truth for the state vector, while ${\hat{x}}_{i}$ and *P* are the estimated state and covariance matrix. Furthermore, the estimated positions of landmarks are also evaluated with RMS, to be shown in Table 2 later. In NEES, $\varepsilon_{i}$ is chi-square distributed with $n_{x}$ degrees of freedom, where $n_{x}$ represents the dimension of *x*. To evaluate the consistency, the average NEES is necessary for *N* Monte Carlo trials:

(35) $$\bar{\varepsilon }=\frac{1}{N}\sum_{i=1}^{N}\varepsilon_{i}$$

**Figure 4:**
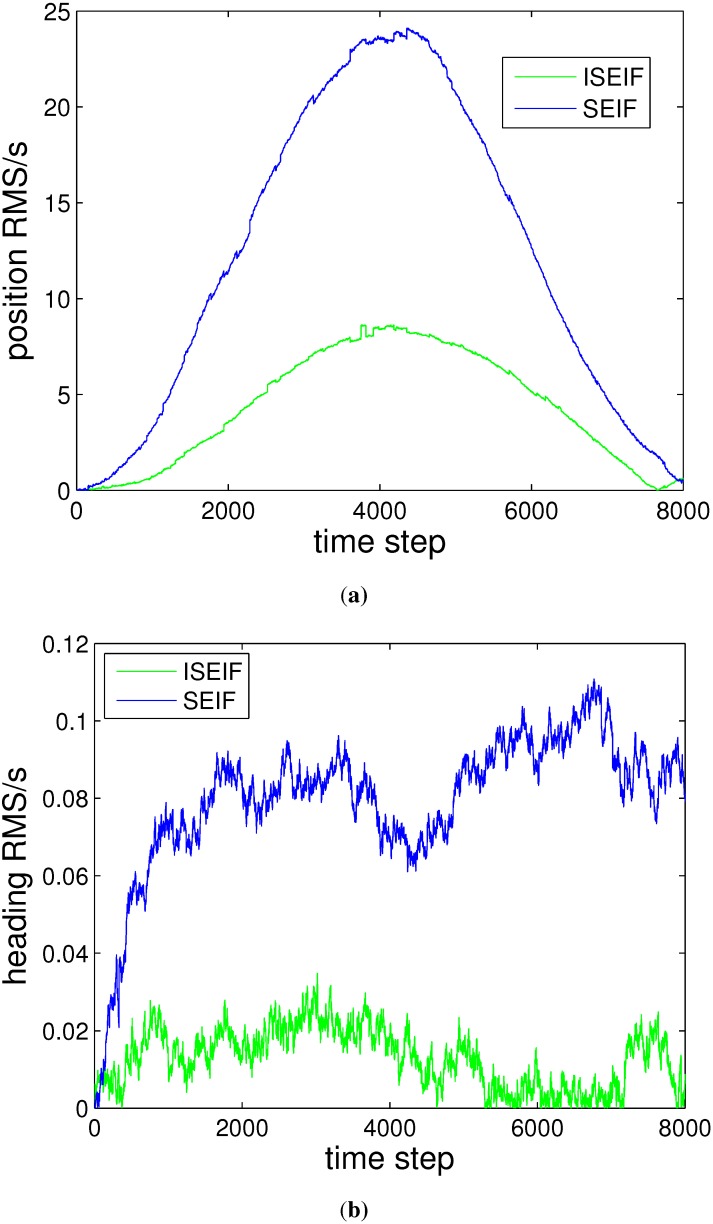
The RMS errors of positions and headings of SEIF (**a**) and ISEIF (**b**).

Figure 4 respectively compares the RMS errors of vehicle positions and the headings of SEIF and ISEIF. Apparently, the trend of errors of ISEIF goes steadily and smaller than that of SEIF, including both vehicle position errors and heading errors. All of the errors decrease by about 80 percent under the action of ISEIF. Besides the superiority of the algorithm itself, the simplicity of simulation data may be another reason that produces so much improvement in the accuracy.

Here, some detailed results calculated by RMS are listed in Table 2. They are average values over 50 independent simulations, only varying by the different random noises that we add. As the list shows, the errors of the parameters that we obtain all decrease by more than 20% after being optimized by ISEIF. Thus, we can draw the conclusion that ISEIF can be much more effective for improving accuracy.

**Table 2:** Average errors over 50 simulations.

	Pose-x Error	Pose-y Error	Heading Error	Landmark-x Error	Landmark-y Error
SEIF	19.9203 m	9.1883 m	$0.0052^{\circ }$	18.9011 m	9.9527 m
ISEIF	15.8803 m	7.6626 m	$0.0031^{\circ }$	14.1157 m	7.6663 m

**Figure 5:**
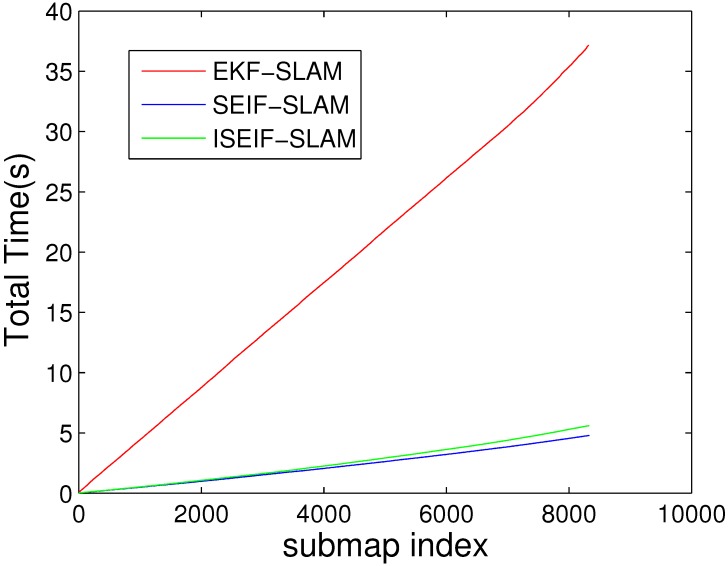
The comparison of average CPU time for EKF, SEIF and ISEIF.

Figure 5 illustrates the average NEES values, where Figure 5a is for SEIF and Figure 5b is for ISEIF. In our simulations, the dimension of the vehicle pose is three, and there are 50 Monte Carlo trials performed; thus, *N* = 50. The 95% probability concentration region for $\bar{\varepsilon }$ confines the horizontal threshold at [2.36, 3.72], produced by the average chi-square distribution [32], as the red lines plotted in the figure. It can be seen that the NEES value of ISEIF keeps close to the bound all of the time, while the result of SEIF goes far away quickly, which persuasively indicates that ISEIF keeps a more consistent estimate.

**Figure 6:**
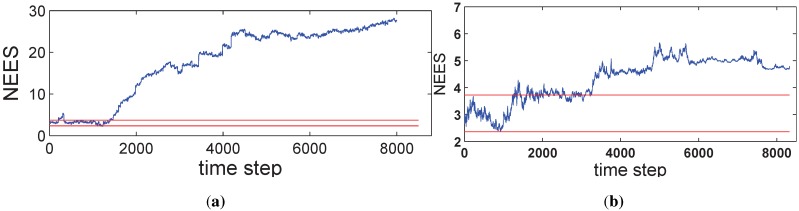
Average normalized estimation error square (NEES) of SEIF (**a**) and ISEIF (**b**).

For the purpose of demonstrating the speed advantage of ISEIF, the total CPU time is presented. The comparison of the average time of 50 Monte Carlo experiments between EKF, SEIF and ISEIF is illustrated in Figure 6. According to the results, the total execution times of EKF, SEIF and ISEIF are 37.1720 s, 4.7845 s and 5.5987 s, respectively. Thus, we can see that compared to EKF, SEIF and ISEIF are dramatically time saving. As for ISEIF, it only consumes a little more time than SEIF, but improves the estimate accuracy and consistency.

### 3.2. Victoria Park Dataset

Different from simulations, real-world applications often involve a variety of uncertainty factors, such as non-Gaussian noise and nonlinear motion and measurement models. To validate the ISEIF and compare the performances of it with the SEIF, we apply them to the benchmark Victoria Park dataset, which is commonly used to test the feasibility of different algorithms in the SLAM community. The environment is approximately 250 m east to west and 300 m north to south.

In the experiment [33], a sensor-enabled car, equipped with odometry sensors and a laser range finder, was driven autonomously in a series of irregular routes within Victoria Park, Sydney.

#### 3.2.1. Sensors

The car used for the experiments is equipped with a SICK (as spelled by a producer of sensors from Germany) laser range and bearing sensor, a linear variable differential transformer sensor for the steering mechanism and a back wheel velocity encoder. The inertial measurement unit consists of three accelerometers, three gyros and two inclinometers, as well as external sensors, including GPS, laser and vision. Here are some pivotal sensors used in the experiment.

GPS provides the data that describe the ground truth, which are comprised of the time stamp, latitude and longitude in meters with respect to the initial point.

The SICK laser scanner provides 360 range points at 0.5-degree intervals. More specifically, it uses 1${}^{\circ }$ to 360${}^{\circ }$ to represent a range at 0${}^{\circ }$ to 180${}^{\circ }$ with an accuracy of 0.5${}^{\circ }$.

#### 3.2.2. Results

**Figure 7:**
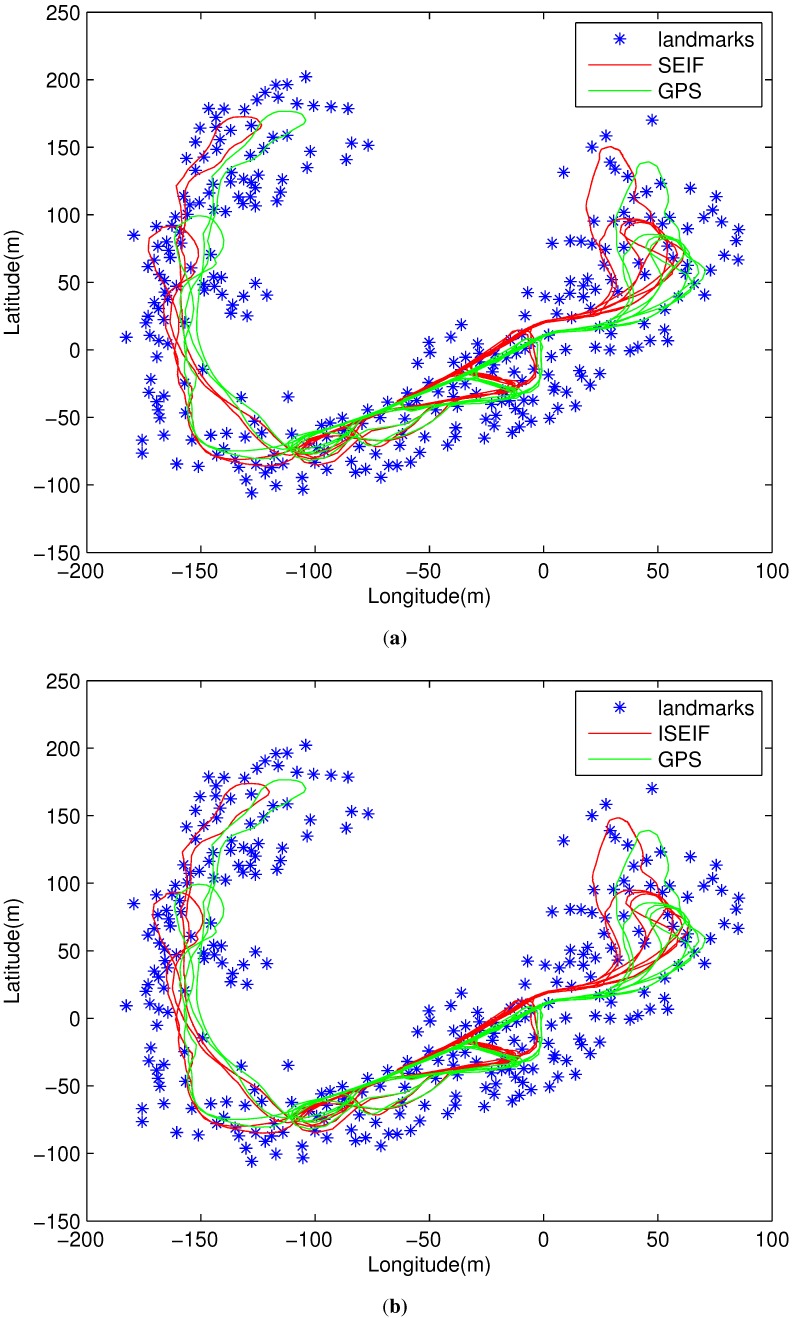
Estimates of the vehicle trajectories and landmarks for the Victoria Park dataset generated by the SEIF (**a**) and the ISEIF (**b**).

The final estimated maps and trajectories of both SEIF and ISEIF are presented in Figure 7a,b. It is obvious that the trajectory estimated by ISEIF is much closer to the GPS, which validates the feasibility of ISEIF.

**Figure 8:**
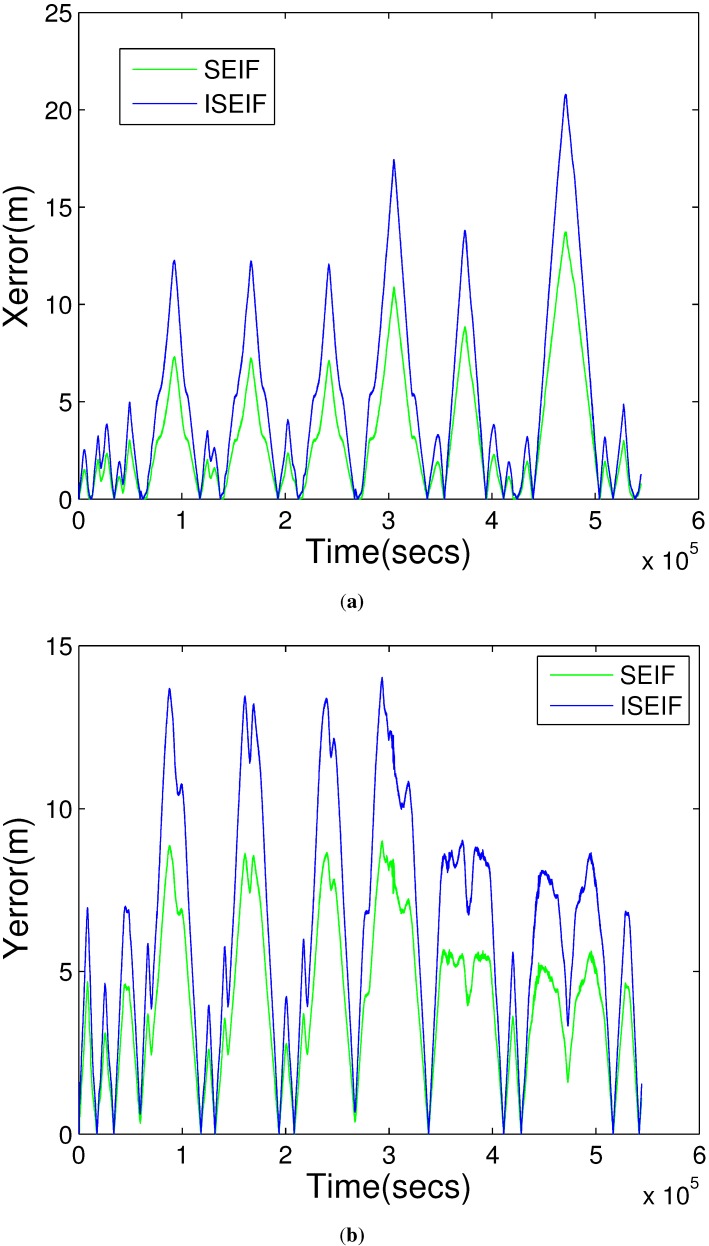
The RMS error comparison between SEIF and ISEIF relative to GPS, where (**a**) is the error in horizontal coordinate and (**b**) is in vertical coordinate.

Further analysis focuses on the accuracy and the consistency of the two algorithms, which are assessed by RMS and NEES, respectively. The plot in Figure 8 displays the RMS errors of SEIF and ISEIF with overlap, which indicates that the ISEIF performs a more accurate estimate than SEIF. It can be seen that the estimated errors grow when the vehicle explores new regions and decrease when the vehicle returns to known areas. ISEIF is more accurate than SEIF at all times.

**Figure 9:**
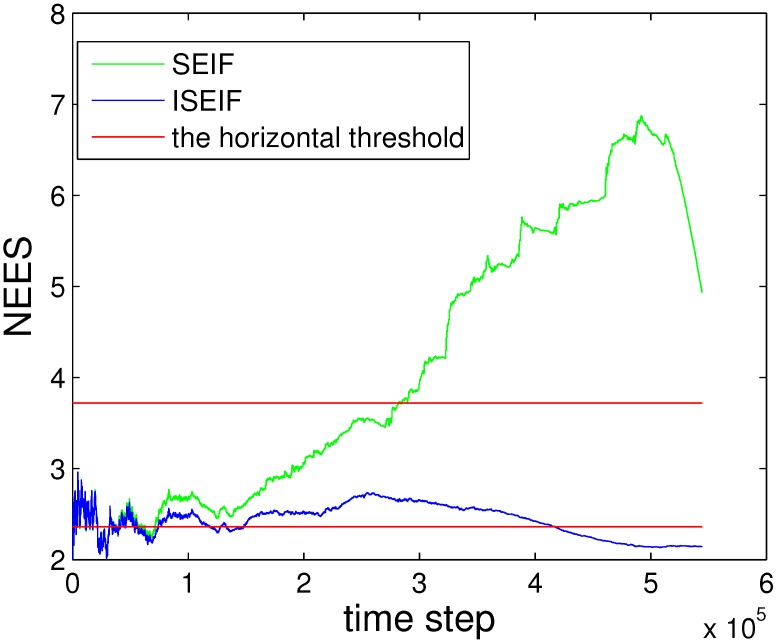
Plots of the NEES of both SEIF and ISEIF for the Victoria Park dataset.

Compared to SEIF, the consistency performance of ISEIF also improves greatly, which can be reflected by NEES in Figure 9. The NEES value of ISEIF is mostly bounded in the threshold interval, while the NEES value of SEIF soon goes beyond the upper threshold.

**Figure 10:**
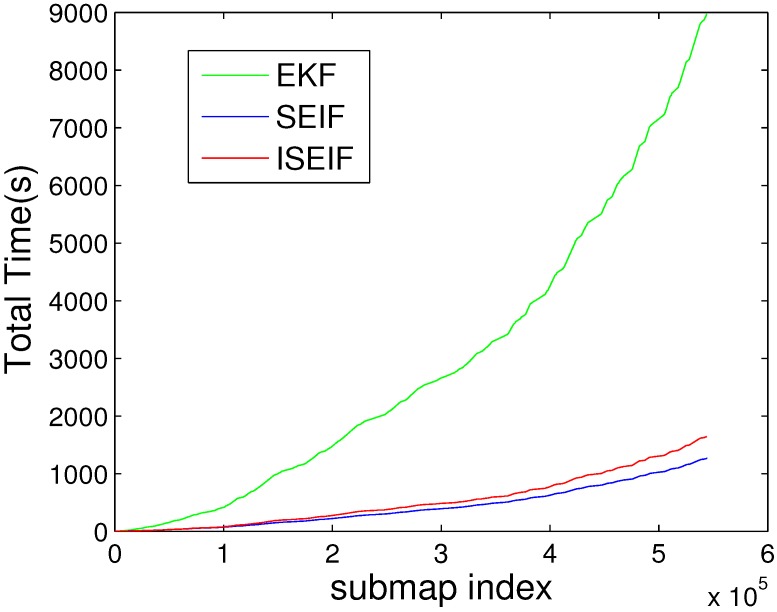
CPU time elapsed for the Victoria Park dataset generated by EKF, SEIF and ISEIF.

Moreover, the computational complexity also should be taken into account. Just as the plot shown in Figure 10, the total time required for the time prediction and measurement update steps for ISEIF is slightly longer, but not a large gap, compared to SEIF. For the implementation of ISEIF, it is inevitable to spend a little time due to the iterative step. However, ISEIF still keeps the speed advantage over EKF, while preserving a more accurate and consistent estimate.

Table 3 quantitatively compares the results of SEIF and ISEIF. The results showed that ISEIF achieves about 40% and 10% improvement in pose-x and pose-y accuracy. In terms of consistency, 82.34% of the NEES value of ISEIF is bounded in the confidence interval, while SEIF is only 52.10%. Although ISEIF is about 20% slower than SEIF, it is much faster than EKF in an order. Based on these results, we can draw the conclusion that ISEIF obtains a more consistent and accurate state estimate in a fast speed.

**Table 3:** Comparisons of the performances of SEIF and ISEIF for the Victoria Park dataset.

Algorithm	Pose-x Error	Pose-y Error	NEES	CPU Elapsed Time
SEIF	7.5284 m	5.3781 m	51.10%	1374.28 s
ISEIF	4.7133 m	4.8325 m	82.34%	1658.31 s

### 3.3. Sea Trials in Tuandao Bay

To verify the feasibility of SEIF more persuasively, we carried out sea trials by an AUV with both SEIF and ISEIF implemented. We will first introduce our experiment platform and analyze the results afterwards.

#### 3.3.1. AUV C-Ranger

**Figure 11:**
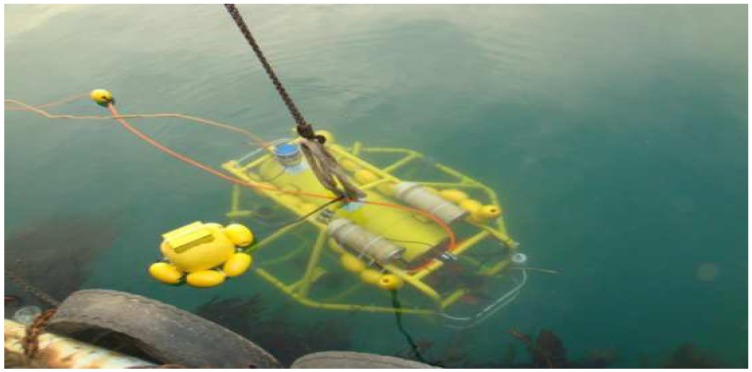
C-Ranger in deployment.

**Figure 12:**
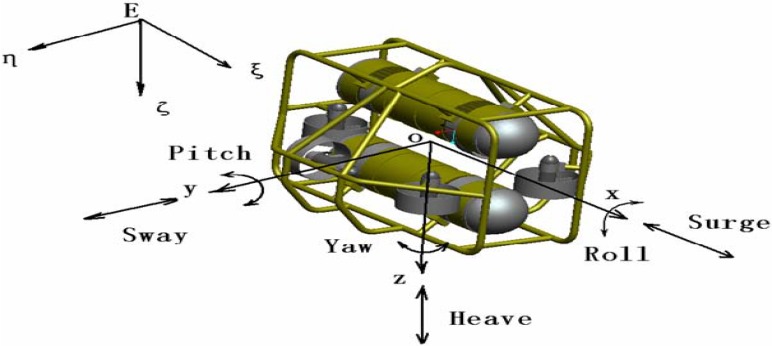
The coordinate system of the C-Ranger platform.

In the following experiments, a C-Ranger AUV loaded with a series of sensors was designed to navigate in underwater circumstances. The C-Ranger is an open-frame AUV, shown in Figure 11, and its general parameters are listed in Table 4. The AUV system is comprised of two electronic cabins and five underwater propeller thrusters. Figure 12 illustrates the coordinate system of the C-Ranger platform. Five degrees of freedom (DOF), including yaw, pitch, roll, heave and surge, contribute to the good maneuverability of the AUV. The thrust system consists of five propeller thrusters. Two of the thrusters paralleling to the bow direction installed in the abdomen are in charge of horizontal thrust related to the surge and yaw, and the other three are vertical thrusters for heave, roll and pitch. The upper hull of the C-Ranger is the instrument compartment containing sensors, the computer module, the communication module, the internal monitoring module and other devices. The lower hull is the power supply system mainly made up of lithium-ion batteries, the power management module, the motor-driver module, *etc*.

**Table 4:** Essential parameters of the C-Range autonomous underwater vehicle (AUV).

Parameter	Length	Width	Height	Tonnage	Weight	Maximal Speed	Endurance
Value	1.6 m	1.3 m	1.1 m	208 L	206 kg (full loaded)	3 knots (1.5 m/s)	8 h at 1 knot speed

#### 3.3.2. On-Board Sensors

There are a lot of sensors installed on the C-Ranger, and they are generally divided into the internal group and the external group. Internal sensors are the digital compass, gyro, the attitude and heading reference system (AHRS) and the pressure sensor. External sensors include mechanical scanning sonar, the Doppler velocity log (DVL), altimeter, CCD camera and GPS. Among them, the mechanically scanning forward-looking sonar (Super Seaking DST, Tritech) for active sensing of environment features installed at the front top of C-Ranger is the principal sensor. It works at a frequency of 675 kHz and a range of up to 300 m.

#### 3.3.3. AUV Motion Model

At time *t*, the control inputs $U^{t}=\left\{u_{1},u_{2}\cdots u_{t}\right\}=\left\{U^{t-1},u_{t}\right\}$ and observations $Z^{t}=\left\{z_{1},z_{2}\cdots z_{t}\right\}=\left\{Z^{t-1},z_{t}\right\}$ are available. The AUV motion model of the experiments is shown as follows:

(36) $$X_{t+1}=\left(\begin{array}{c} x_{vt}\\ y_{vt}\\ \phi_{vt} \end{array}\right)=\left(\begin{array}{c} x_{vt}+\Delta TV\cos\left(\theta +\theta_{vt}\right)\\ y_{vt}+\Delta TV\sin\left(\theta +\theta_{vt}\right)\\ \theta_{vt}+\frac{\Delta TV\sin\theta }{L} \end{array}\right)+v_{t}$$

where the variables L,V,θ,ΔT represent vehicle length, vehicle velocity, rotation angle and sampling interval, respectively. $v_{t}∼N\left(0,Q\right)$ is white Gaussian noise. Correspondingly, the AUV observation model related to the range and bearing is:

(37) $$z_{t}=\left(\begin{array}{c} z_{r}\\ z_{\alpha } \end{array}\right)=\left(\begin{array}{c} \sqrt{{\left(x_{i}-x_{vt}\right)}^{2}+{\left(y_{i}-y_{vt}\right)}^{2}}\\ \arctan\left(\frac{y_{i}-y_{vt}}{x_{i}-x_{vt}}\right)-\theta_{vt} \end{array}\right)+\left(\begin{array}{c} w_{rt}\\ w_{\alpha t} \end{array}\right)$$

${\left(x_{i},y_{i}\right)}^{T}$ is the global position of *i*-th feature and $w_{rt}$ and $w_{\alpha t}$ are the observation Gaussian noise, namely $w_{rt}∼N\left(0,R_{r}\right)$, $w_{\alpha t}∼N\left(0,R_{\alpha }\right)$.

#### 3.3.4. Sea Trial

**Figure 13:**
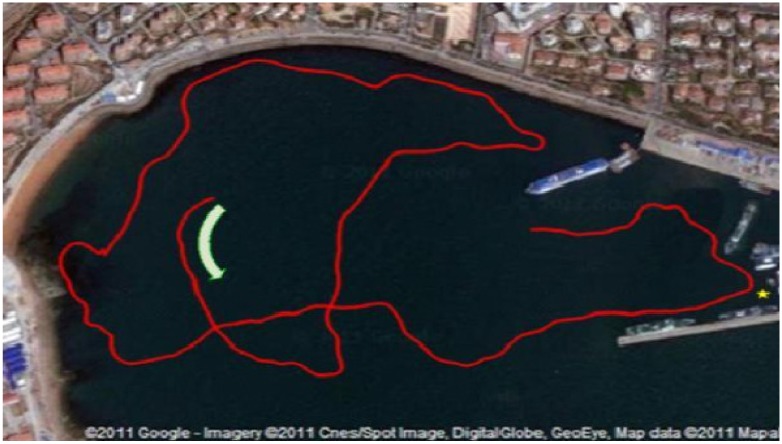
The satellite map of Tuandao Bay and the trajectory of C-Ranger by GPS.

The environment where we conducted our sea trials is as shown in Figure 13, which is a satellite map of Tuandao Bay in Qingdao, China, added with the GPS trajectory along which the vehicle moves (the red line). The trajectory of the GPS is considered as the ground truth as in the simulation. In the experiment, the vehicle moves at a speed of about one knot; the imaging sonar is set to a 120-degree field of vision; and the scanning range is set to 100 m.

**Figure 14:**
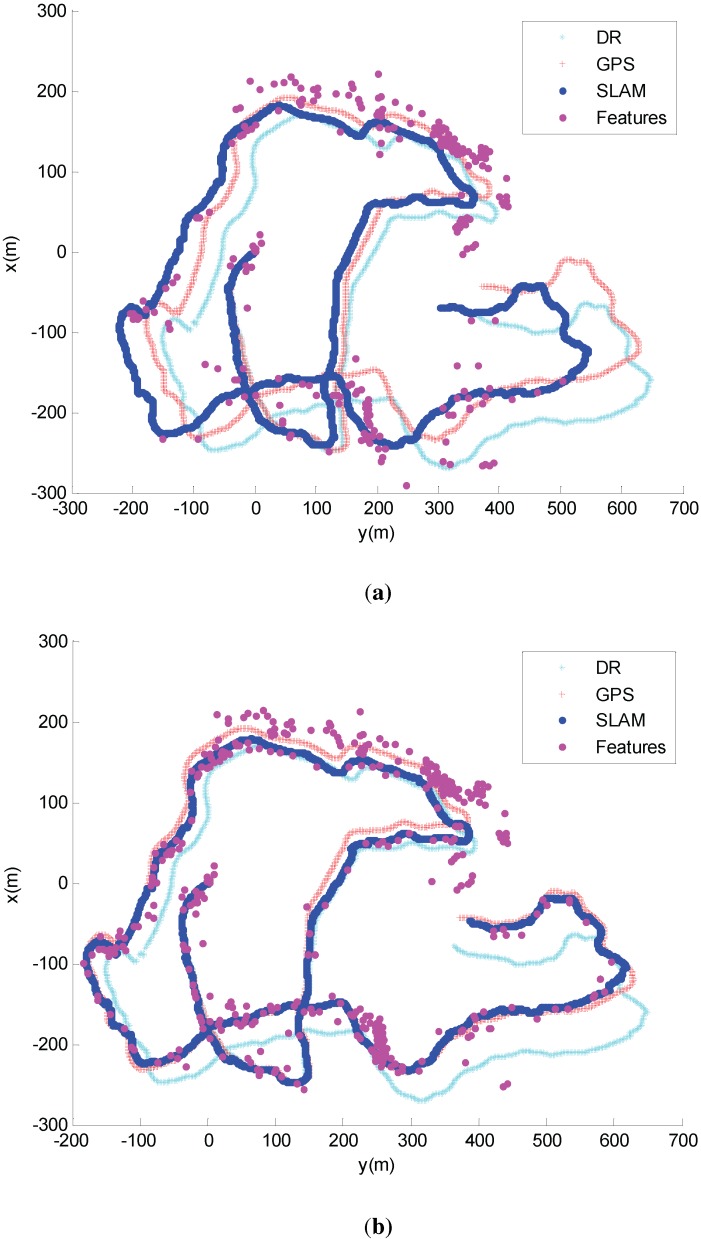
Comparisons of the results estimated by SEIF (**a**) and ISEIF (**b**) in the experiments of the sea trial.

The comparison result of the trajectories is illustrated in Figure 14, where the red line represents the GPS trajectory, the light blue is for dead reckoning and the dark blue is for the SLAM. Here, Figure 14a,b shows the result of SEIF and ISEIF, respectively. As the figures indicate, the trajectory estimated by ISEIF fits the GPS trajectory better than that of SEIF. Owing to the great amount of sonar data, there exist some redundant features inevitably. Thus, raw data measured by sonar are preprocessed to reduce the computation, and the feature extraction algorithm is the same as in [29].

**Figure 15:**
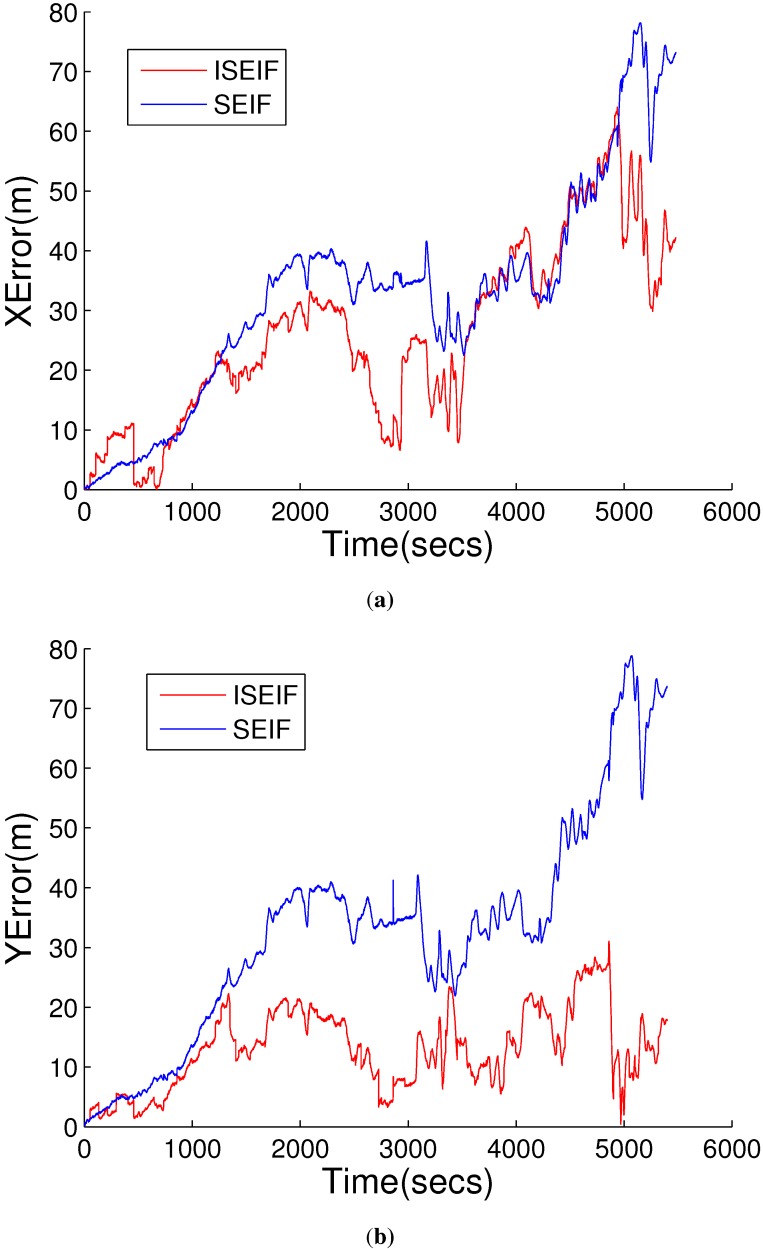
The RMS error comparison between SEIF and ISEIF relative to GPS, where (**a**) is the error in horizontal coordinate and (**b**) is in vertical coordinate.

Furthermore, the RMS error is used to compare the accuracy of the two algorithms quantitatively. Figure 15a,b presents the RMS errors of horizontal and vertical coordinates, respectively. The figures show that the RMS errors of ISEIF have a similar trend to SEIF, but much smaller than SEIF in general, both in horizontal and vertical coordinates. As is shown in Figure 8 and Figure 15, the decrease of the RMS errors of real experiments is much smaller than that of simulations. This can be explained by the inevitable performance reduction of ISEIF during processing complex and large datasets. Despite this, all experiments still validate the effectiveness of ISEIF on improving navigation accuracy. Thus, we can draw the conclusion that ISEIF performs more accurate estimates in real-world environments.

**Figure 16:**
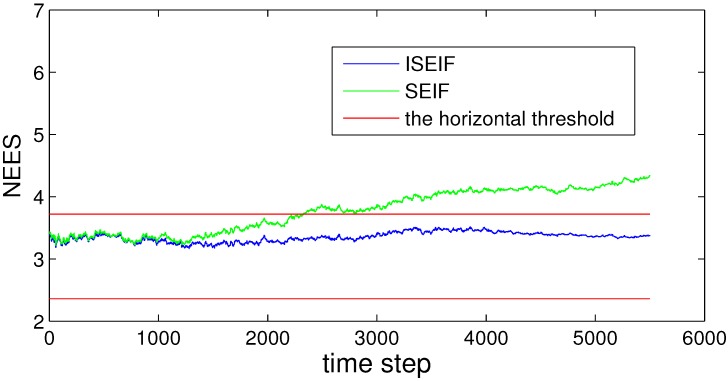
The comparison of NEES for SEIF and ISEIF in the sea trial.

Compared to SEIF, ISEIF also shows superior performance in terms of consistency, which can be reflected apparently by NEES in Figure 16. The NEES value of ISEIF is mostly bounded in the threshold interval, while the NEES value of SEIF soon goes beyond the upper threshold. According to Figure 4, Figure 9 and Figure 16, NEES of ISEIF can basically keep close to the threshold and maintain a relatively lower value steadily, while the corresponding NEES of SEIF tends to deviate far away from the threshold. In summary, ISEIF can effectively optimize consistency.

**Figure 17:**
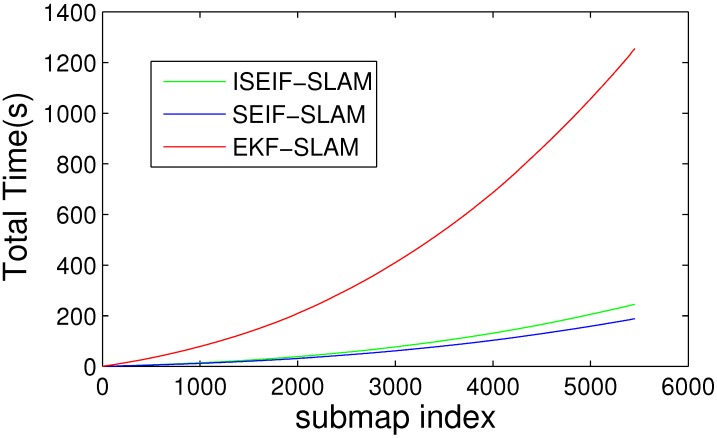
The comparison of CPU time for EKF, SEIF and ISEIF in the sea trial.

The elapsed CPU times of EKF, SEIF and ISEFI were also recorded, and Figure 17 shows the results. It is very similar to the simulations we have done before, *i.e.*, ISEIF also shows a speed advantage in real-world applications. Again for the reasons of complex data and an unstable environment, many divergences emerge. This leads to the increase of iterative operations, which makes the running time of the sea trial extended for more that tens of seconds. Even so, the total time can be still considered acceptable and fairly fast for ISEIF compared to traditional EKF. For such a large dataset, this extra time is worth spending to obtain more consistent results. In conclusion, the experimental results confirm the excellent performance of ISEIF in both accuracy and efficiency.

Table 5 quantitatively shows the results of SEIF and ISEIF in the sea trials. From the results, we can see that ISEIF achieves about 25% and 70% improvement in pose-x and pose-y error, respectively. In terms of consistency, 100% of the NEES value of ISEIF is bounded in the confidence interval, while SEIF is only 48.23%. Although ISEIF is about 35% slower than SEIF, it is still much faster than EKF. Then, we can draw a similar conclusion as in simulations and the Victoria Park dataset. It should be noted that as the simulated experiments are much simpler than real-world environments, it is almost a sure thing that our new method performs best in simple simulations. In fact, real-world environments are always very complex, and many factors might influence the results (e.g., the quality of the data). Therefore, it is more difficult to handle real-world applications. However, ISEIF still shows better consistency and accuracy compared to SEIF and EKF with a fast enough speed. In our future work, we will continue to optimize our implementation of the algorithms and the AUV platform.

**Table 5:** Comparisons of performances of SEIF and ISEIF for the sea trial.

Algorithm	Pose-x Error	Pose-y Error	NEES	CPU Elapsed Time
SEIF	36.4628 m	40.7963 m	48.23%	285.23 s
ISEIF	27.7952 m	12.1165 m	100%	199.58 s

## 4. Discussion

Speed and consistency are the two main issues in the SLAM research community [34,35,36]. This paper focuses on the nonlinearity of the SLAM problem to improve the consistency. The proposed algorithm can reduce the linearized error by iteration and then get a more conservative estimate. It can also keep a fast speed at the same time.

Actually, both EKF and SEIF can be classified into one kind of method, *i.e.*, parametric filters, which belong to the online SLAM family. They are all based on the same probabilistic framework:

(38) $$\underset{s_{t},m}{\arg\max}P\left(s_{t},m|z_{1:t},u_{1:t}\right)$$

which can be thought of as calculating the most likely current pose $s_{t}$ and the map *m* based on the controls and the measurements.

The fundamental reason for the inconsistency problem arising from the parametric filter may be that it only maintains the current pose, not the entire trajectory. Without the entire trajectory, the filter can only use new information to correct the current pose, not all of the previous pose. Thus the accumulated error cannot be revoked. In addition, the approximation of the nonlinearity in the SLAM problem is another reason for inconsistency. Actually, this paper only addresses the second part listed above, while how to reduce accumulated error was not discussed. Future work should be focused on this problem.

At the current time, full SLAM, also known as smoothers, shows its popularity in SLAM research community [37,38,39,40,41,42]. It is based on the following probabilistic formulation:

(39) $$\underset{s_{t},m}{\arg\max}P\left(s_{t},m|z_{1:t},u_{1:t}\right)$$

The subtle difference of full SLAM is that it estimates the entire trajectory of the robot, not only the current pose. Although full SLAM shows its popularity in the SLAM research community, there still exist some bottlenecks which limit its application. Among them, the most important one is that full SLAM algorithms are always more complex than parametric filters, thus implementing it in practical systems is difficult. That is why we still improve the SEIF algorithm, which is very easy to implement in real-world applications. In the future, we will also try to implement the full SLAM algorithms in engineering applications.

## 5. Conclusions

In this paper, the iterative sparse extended information filter (ISEIF) has been proposed to improve the consistency of SEIF. As a dual form of the EKF, the extended information filter is subject to the same consistency problem. SEIF tends to be more overconfident than EIF due to the sparsification and state recovery step. Hence, the iterative method was adopted to get more conservative estimates. Mathematical derivation of ISEIF was given in this paper, and the effect of iteration during the update process was illustrated. To demonstrate the performance of ISEIF, Monte Carlo simulations were conducted, and both RMS and NEES errors were presented. The results convincingly show that ISEIF performs a more conservative estimate than SEIF. The time costs of EKF, SEIF and ISEIF were also compared. The results indicate that although ISEIF is a little slower than SEIF, it still keeps the scalability advantage over EKF, making it applicable in large environments. Real-world environments are very different from simulated experiments. Therefore, we also verified the validity of ISEIF in both outdoor and underwater navigation. The experimental platform AGV and the C-Ranger AUV were presented first, and experiments were conducted in Victoria Park and Tuandao Bay, respectively. Results empirically show that ISEIF is both consistent and scalable.
